# Delayed gametocyte clearance in *Plasmodium vivax* malaria is associated with polymorphisms in the cytochrome P450 reductase (CPR)

**DOI:** 10.1128/aac.01204-23

**Published:** 2024-02-27

**Authors:** Yanka Evellyn Alves Rodrigues Salazar, Jaime Louzada, Maria Carolina Silva de Barros Puça, Luiz Felipe Ferreira Guimarães, José Luiz Fernandes Vieira, André Machado de Siqueira, José Pedro Gil, Cristiana Ferreira Alves de Brito, Tais Nobrega de Sousa

**Affiliations:** 1 Molecular Biology and Malaria Immunology Research Group, Instituto René Rachou, Fundação Oswaldo Cruz (FIOCRUZ), Belo Horizonte, Minas Gerais, Brazil; 2 Universidade Federal de Roraima, Boa Vista, Roraima, Brazil; 3 Universidade Federal do Pará, Belém, Pará, Brazil; 4 Instituto Nacional de Infectologia Evandro Chagas, Fundação Oswaldo Cruz (FIOCRUZ), Rio de Janeiro, Rio de Janeiro, Brazil; 5 Department of Microbiology, Tumor and Cell biology, Karolinska Institutet, Solna, Sweden; The Children's Hospital of Philadelphia, Philadelphia, Pennsylvania, USA

**Keywords:** malaria, *Plasmodium vivax*, gametocytes, primaquine, pharmacogenetics, cytochrome P450 reductase, CYP2D6

## Abstract

Primaquine (PQ) is the main drug used to eliminate dormant liver stages and prevent relapses in *Plasmodium vivax* malaria. It also has an effect on the gametocytes of *Plasmodium falciparum*; however, it is unclear to what extent PQ affects *P. vivax* gametocytes. PQ metabolism involves multiple enzymes, including the highly polymorphic CYP2D6 and the cytochrome P450 reductase (CPR). Since genetic variability can impact drug metabolism, we conducted an evaluation of the effect of CYP2D6 and CPR variants on PQ gametocytocidal activity in 100 subjects with *P. vivax* malaria. To determine gametocyte density, we measured the levels of *pvs25* transcripts in samples taken before treatment (D0) and 72 hours after treatment (D3). Generalized estimating equations (GEEs) were used to examine the effects of enzyme variants on gametocyte densities, adjusting for potential confounding factors. Linear regression models were adjusted to explore the predictors of PQ blood levels measured on D3. Individuals with the *CPR* mutation showed a smaller decrease in gametocyte transcript levels on D3 compared to those without the mutation (*P* = 0.02, by GEE). Consistent with this, higher PQ blood levels on D3 were associated with a lower reduction in *pvs25* transcripts. Based on our findings, the *CPR* variant plays a role in the persistence of gametocyte density in *P. vivax* malaria. Conceptually, our work points to pharmacogenetics as a non-negligible factor to define potential host reservoirs with the propensity to contribute to transmission in the first days of CQ–PQ treatment, particularly in settings and seasons of high *Anopheles* human-biting rates.

## INTRODUCTION

*Plasmodium vivax* is the most prevalent malaria species in the Americas, responsible for approximately 89% of the 160,000 cases annually reported in Brazil in the last 4 years (1). The highest incidence rates occur in the Legal Amazon region, which corresponds to 59% of the Brazilian territory in the country’s northern region (2).

During the malaria parasite’s life cycle, the sexual blood stage gametocytes link the vertebrate host and the vector, making them prime targets for intervention strategies aiming at blocking transmission. The lifespan of gametocytes is reduced by the action of primaquine (PQ) in infections caused by *P. falciparum* (3–5). Similarly, the gametocyte clearance time is significantly shorter when PQ is added to chloroquine to treat patients with *P. vivax* malaria (6). In addition, children who received PQ treatment in Papua New Guinea had a lower chance of becoming positive for *P. vivax* gametocytes than those who did not receive PQ (7).

PQ is a tissue schizonticide that needs to be bio-transformed into an active metabolite, i.e., it represents a prodrug that needs to be metabolized to generate molecules that exert hypnozoiticidal and gametocytocidal activity (8, 9). Hypnozoites are latent stages of the parasite that remain dormant in hepatocytes, leading to recurrence of symptoms when active (10). The PQ metabolism is rapid after drug ingestion, reaching peak plasma levels within 2–3 hours and then rapidly declining with a terminal elimination half-life between 4 and 9 hours (11, 12).

The mechanism of action of PQ is still unclear, but the involvement of CYP2D6 in PQ metabolism is well-established (8, 9). Pharmacokinetic studies of PQ have demonstrated its metabolic dependence via the CYP2D6 pathway, indicating that the presence of polymorphisms in the gene can alter the rate of PQ metabolism (13–15). The *CYP2D6* gene is highly polymorphic, with over 130 alleles associated with variable phenotypes ranging from complete dysfunction to ultra-rapid metabolism (16, 17). The decreased enzymatic function associated with diminished prodrug activation may lead to increased clinical recurrences of malaria as the hypnozoite clearance is compromised. The metabolism of PQ by CYP2D6 occurs in a joint action between the CYP system and its substrates, including the electrons provided by the cytochrome P450 reductase enzyme (CPR) (18, 19). Mutations present in or near flavin adenine dinucleotide/flavin mononucleotide (FAD/FMN) domains can result in severe effects on CPR activity, which may affect metabolic processes significantly dependent on CYPs (20, 21). The most common A503V *CPR* polymorphism, located near the FAD domain, defines the CPR*28 allele. It presents a moderate effect (40%–50%) on CYP2D6 activity, possibly contributing to genetic variability in drug and xenobiotic metabolism (22).

PQ is converted into hydroxylated metabolites (OH-PQm) with consequent hydrogen peroxide (H_2_O_2_) formation, which accumulates at sites of metabolic transformation, leading to antiparasitic action (23, 24). PQ activity against *P. falciparum* gametocytes is significantly enhanced by direct reduction of OH-PQm by the complex CPR (24). A few studies have addressed the direct impact of CYP2D6 enzyme activity on the gametocytocidal effect of PQ in malaria. A retrospective study on African populations treated with a single dose of PQ (0.1 to 0.75 mg/kg) combined with artemisinin-based combination therapy (ACT) showed longer *P. falciparum* gametocyte clearance after treatment of patients with impaired CYP2D6 activity (25). On the other hand, another clinical trial conducted in Tanzania demonstrated that single-dose PQ treatment was sufficient to reduce *P. falciparum* gametocyte levels regardless of the CYP2D6 status (26).

The association between the CPR/CYP2D6 complex and gametocyte clearance has been investigated only in *P. falciparum* infections (24). Here, we further explored the effects of polymorphisms in the CPR/CYP2D6 complex on gametocyte clearance of *P. vivax* by conducting a 3-day follow-up study of individuals with *P. vivax* malaria from the Brazilian Amazon region. We hypothesized that mutations in this enzymatic complex could diminish the effectiveness of PQ, resulting in a delay in gametocyte clearance.

## RESULTS

One hundred adults with a median age of 33 years (IQR, 26–42) were enrolled (Table 1). Most of them were males who possibly became infected in gold mining areas in the north region of Brazil. They are found to frequently migrate due to mining activities. Almost 40% experienced at least one episode of *P. vivax* malaria recurrence in 6 months.

**TABLE 1:** Demographic and genetic characteristics of subjects enrolled in this study

Characteristics	Total population
Age, median (IQR, years)	33 (26–41.5)
Gender, n (%)	
Male	80 (80)
Female	20 (20)
Possible area of infection, n (%)	
Gold mining	63 (63)
Others	37 (37)
Number of malaria recurrence episodes, n (%)	
0	61 (61)
1	23 (23)
>2	16 (16)
CYP2D6 activity score, n (%)**^*a*^**	
<1.0	30 (30)
>1.0	67 (67)
CPR A503V, n (%)**^*b*^**	
CC	65 (65)
CT	28 (28)
TT	7 (7)

^*a*^ Three participants were not included in the analyses due to an indetermined phenotype.

^*b*^ 1508C > T variant.

All subjects contributed with two blood samples before drug treatment (D0) and 72 hours (D3) after a standard CQ and PQ treatment regime. They were successfully genotyped for polymorphisms in CYP2D6 (97%) and CPR (100%). Among them, 30% had impaired enzyme activity for CYP2D6 as inferred from genotype data (AS ≤1.0) (Table 1; Table S1). There was no association between recurrence and CYP2D6 activity [27 out of 67 (40%) had one or more episodes of recurrence in the AS >1.0 group versus 10 out of 30 (33%) in the AS ≤1.0 group, *P* = 0.514 by the chi-squared test]. Also, the time to the first recurrence was not influenced by the CYP2D6 status (*P* = 0.423, Kaplan–Meier log-rank test). However, it is important to consider that most individuals exerted activities related to gold mining, where malaria transmission is high. Thus, one could not exclude the possibility of an infection due to a new mosquito bite.

We also evaluated the amino acid substitution A503V in CPR, which has been previously shown to reduce CYP2D6 activity. Thirty-five subjects carried the mutated allele in homo- (7%) or heterozygosity (28%). The CPR status was not associated with the number of recurrence episodes of *P. vivax* malaria (34% of carriers of the mutated allele in homo- or heterozygosity vs 42% without mutation experienced *P. vivax* recurrences; *P* = 0.525, by Fisher’s exact test).

**Fig 1:**
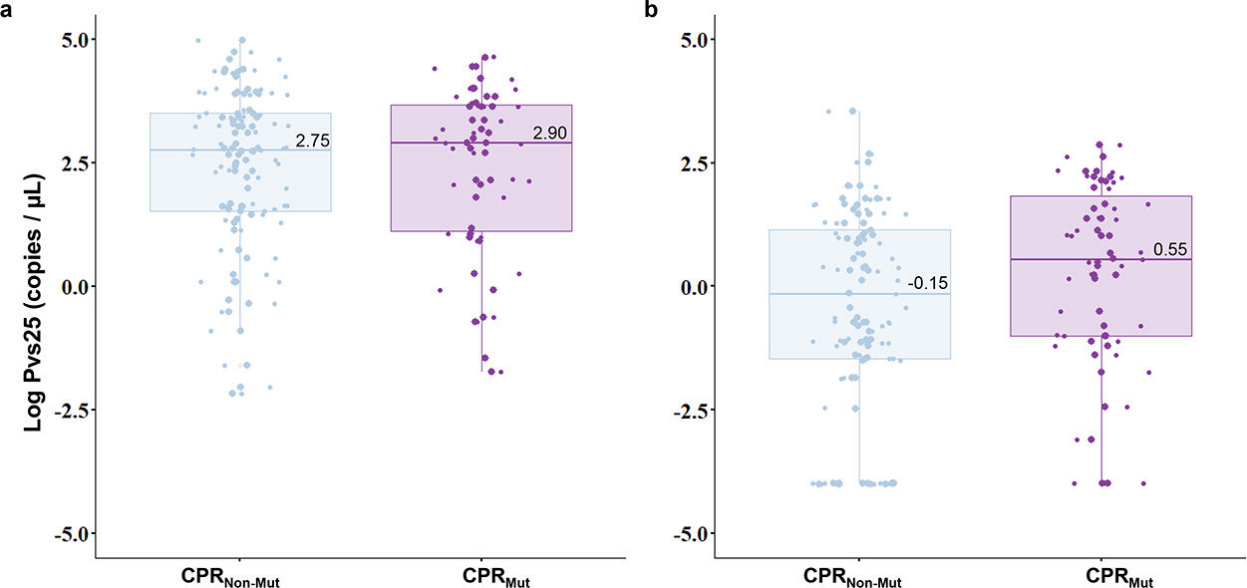
Gametocyte density estimates according to the cytochrome P450 reductase (CPR) status. Gametocyte density is shown as the log of *pvs25* transcript levels (**A**) at baseline and (**B**) 72 hours after the initiation of the treatment. According to the conversion of *pvs25* transcript levels into numbers of gametocytes published by Koepfli and colleagues (27) (1 gametocyte = 4.17 pvs25 transcripts), the median number of gametocytes is 134.8 [(IQR, 7.35–825.6) for CPR_Non-Mut_ and 191.5 (IQR, 2.82–1,217)] for CPR_Mut_ at baseline and 0.06 (IQR, 0.0–3.09) for CPR_Non-Mut_ and 0.85 (IQR, 0.02–23.1) for CPR_Mut_ on D3. CPR_Non-Mut_, CPR non-mutated; CPR_Mut_, CPR mutated (homo and heterozygous).

We next evaluated how PQ metabolism was associated with gametocyte density. The gametocyte positivity and density were assessed by quantifying *pvs25* transcripts. Ninety-five percent of individuals presented with *pvs25* transcripts at baseline (D0) and 77% on D3. The average levels of the *pvs25* transcript were similar on D0 irrespective of the CPR status (Fig. 1A). By adjusting a longitudinal regression model GEE controlling for the effect of potentially confounding variables, we found that subjects with CPR mutations had gametocyte levels 11.5 times higher on average on D3 than individuals without mutation in CPR (95% CI 1.2–55.2, *P* = 0.013) (Fig. 1B; Table S2). The median number of gametocytes on D3 could be roughly estimated as 0.06 (IQR, 0.0–3.09) and 0.85 (IQR, 0.02–23.1) for CPR non-mutated and mutated subjects, respectively. The total parasitemia determined by the quantification of *18 s rRNA* transcripts was also significantly associated with the levels of the *pvs25* transcript over time. A similar trend regarding gametocyte densities on D3 was seen for CPR A503V genotypes (Fig. S1).

### Primaquine blood level is associated with gametocyte densities

We evaluated whether the concentration of PQ in the whole blood was related to the CYP2D6 and CPR status, as well as to the gametocyte densities over time. Although the PQ concentration had high variability, it was associated with the predicted CYP2D6 phenotype (Fig. S2). PQ levels for carriers of the CPR mutated allele and impaired CYP2D6 activity showed a median of 197.5 ng/mL (IQR, 103–367) while individuals with normal activity of CYP2D6 presented a median of 167.5 ng/mL (IQR, 27–249). By fitting a linear regression model, we found evidence for carriers of CPR mutated allele showing higher dosage of PQ in the presence of impaired CYP2D6 activity when compared to individuals with normal activity of CYP2D6 (β = 66.6, 95% CI 8.5–125, *P* = 0.024) (Table S3). It is noteworthy that a lower reduction of *pvs25* transcript levels between D3 and D0 was associated with a higher dosage of PQ on D3 (*P* = 0.010). Surprisingly, the dosage of PQ was lower for subjects with the CYP2D6 normal phenotype carrying the CPR mutated allele when compared with the subjects without mutation in both enzymes (β = −47.5, 95% CI −91.0–4.1, *P* = 0.036).

## DISCUSSION

Several factors can affect the potential of an infected individual to transmit malaria parasites to mosquitoes, such as gametocyte maturity, the ratio of female to male gametocytes, as well as the human host immunity levels (5). Here, we addressed how impairment of PQ metabolism might affect gametocyte clearance in *P. vivax* infection, which can contribute to maintaining human infection reservoirs. This is an area that still needs to be explored in appropriate depth, particularly in *P. vivax* malaria.

We identified a direct association between CPR status and gametocyte density at 72 hours after PQ–CQ treatment initiation by analyzing polymorphisms in main enzymes related to PQ metabolism. Carriers of polymorphism in *CPR* had a lower reduction in gametocyte density on day 3 compared to non-mutated individuals. This effect was confirmed after considering the initial total parasitemia, which has already been described to influence gametocyte densities (24, 28, 29). These findings agree with those of Camarda and colleagues (24), who observed that *P. falciparum* gametocytocidal activity *in vitro* was greater when in the presence of the active CPR enzyme. CPR has a direct role in the redox cycling of OH-PQm produced by the complex CPR/CYP2D6. The OH-PQm undergo spontaneous oxidation to quinoneimines, leading to the production of H_2_O_2_. Quinoneimines in turn can be reduced back to hydroxylated forms by CPR, resulting in the accumulation of H_2_O_2_, significantly enhancing PQ gametocytocidal activity (24). On the other hand, the A503V variant, despite its highly conservative change and its location near the protein’s surface, away from the FAD-binding site, exhibits reduced catalytic activity (30). Thus, our results provide further evidence for a role of the CPR variant in interindividual differences in response to PQ treatment.

Besides the direct role of CPR in generating H_2_O_2_ and parasite killing, this enzyme is a CYP2D6 redox partner (8, 24). *In vitro* experiments support that hydroxylated PQ metabolites produced through the CPR/CYP2D6 complex exert *per se* modest activity against gametocyte stages, confirming that PQ biotransformation is required for gametocyte killing activity (24). Consistent with that, individuals with impaired CP2D6 activity were associated with prolonged *P. falciparum* gametocyte carriage after treatment (31). Here, we did not find evidence of an association between predicted CYP2D6 enzymatic activity and gametocyte clearance. In the meantime, it is important to stress that only a few individuals were classified as poor (gPM, 2%) or intermediate (gIM, 3%) metabolizers, which restricted us from assessing the effect of these more extreme phenotypes on gametocyte clearance. Here, most subjects with impaired CYP2D6 were classified as normal–slow metabolizers (gNM-S), a phenotype group that shows enzymatic activity between gIM and normal–fast metabolizers (gNM-F) (16). For now, we cannot exclude the possibility that PQ metabolites generated by gNM-S metabolizers through the CPR/CYP2D6 pathway are converted into H_2_O_2_ via CPR-mediated redox cycling exerting effective gametocytocidal activity. Another possible explanation for this lack of association is that CPR is not the exclusive electron donor for CYP2D6 (32, 33). Other pathways may play a role in the process and compensate for alterations in CPR, allowing CYP2D6 to sustain its function during gametocyte clearance.

Our findings were corroborated by the analysis of the PQ concentration in the blood with clear evidence for lower reduction in *pvs25* transcripts among individuals with higher concentrations of PQ on day 3 of drug administration. Consistent with these results, carriers of the CPR mutated allele showed a higher dosage of PQ in the presence of impaired CYP2D6 activity compared with the normal CYP2D6 activity. A clear difference in PQ blood levels was also observed for poor metabolizers showing higher concentrations of the drug and reduced gametocyte clearance on D3. Our study has a limitation because we could not follow a standard approach related to the time of blood collection after drug intake, which may have contributed to the high variability observed in drug blood levels among subjects. Despite that, our results corroborate with those of previous studies showing higher plasma drug levels in gPM/gIM metabolizers and that monitoring blood levels of PQ during treatment can provide a reliable assessment of the parasite exposure to the drug (34, 35). This finding prompts us to inquire whether antimalarial therapy optimization could enhance patient outcomes. However, optimizing drug dosing necessitates a thorough understanding of the drug’s pharmacokinetic and pharmacodynamic properties, along with insights into its metabolizing enzymes. Additionally, achieving optimization should ideally maximize cost–benefit (36). Hence, it becomes imperative to evaluate these conditions in more extensive studies.

This is the first study to evaluate how the CPR/CYP2D6 genetic variability affects the clearance of *P. vivax* gametocytes, highlighting the importance of understanding how pharmacogenetic factors influence the risk of malaria transmission. Specifically, we found that during standard CQ-PQ treatment, the median number of gametocytes on D3 was significantly higher among CPR mutated allele carriers. Of importance, the median of 0.9 gametocytes/µL found among these patients is in the range of the recently determined lowest infective *P. vivax* gametocyte density: 0.2 to 5 gametocytes/µL, with a median of 0.8 gametocytes/µL (37). This contrasts with the significantly lower median of 0.06 gametocytes/µL reported for the CPR wild-type carriers, a value markedly below the documented lower limit for effective infectivity. In operational terms, this suggests that CPR 503V carriers are likely to be still contributing to transmission 72 hours after treatment initiation, while the wild-type–harboring subjects are expected in most cases not to. This fits previous reports of *P. vivax* infectivity studies which showed that, although at much lower rates, asymptomatic individuals with low parasitemia were still able to infect mosquitoes from the Brazilian Amazon (38). Conceptually, our work points to pharmacogenetics as a non-negligible factor to define potential host reservoirs with the propensity to contribute to transmission during CQ–PQ treatment for 3 days, particularly in settings and seasons of high *Anopheles* human-biting rates.

## MATERIALS AND METHODS

### Study site and subjects

To evaluate gametocyte clearance during treatment with PQ–CQ of *P. vivax* malaria, 100 patients were enrolled between October 2019 and March 2020. Eligible subjects were patients with symptomatic *P. vivax* infection of either sex, aged >12 years, attending the government-run malaria clinic Policlínica Cosme e Silva in Boa Vista city. Boa Vista is the capital of Roraima State, in the North Region of Brazil, with about 437,000 inhabitants. According to the Epidemiological Surveillance System for Malaria (SIVEP-Malaria), around 30,000 cases were recorded in 2020, most of them autochthonous cases caused by *P. vivax* (80%). Although Boa Vista is considered a low-risk area for malaria transmission, many cases from other regions have been recorded in recent years (39). In addition, the region and neighboring countries’ surroundings have mining areas in which working conditions are considered a significant problem for disease control. Since 2017, Roraima has experienced intense migration of the Venezuelan and Guyanese population, which has affected malaria control measures and contributed to the spread of the disease in the state (39).

The enrolled participants were diagnosed with a mono-infection by *P. vivax* through microscopic examination of Giemsa-stained thick blood smears evaluated by well-trained microscopists following the malaria diagnosis guidelines of the Brazilian Ministry of Health. Subsequently, a non-ribosomal qPCR assay was conducted for the purpose of molecular diagnosis, targeting Pvr47/Pfr364 as described in a previous study (40). The prescribed treatment for the subjects followed the guidelines of the Ministry of Health, which consisted of a combination of CQ and PQ. The total dosage for CQ was 25 mg/kg administered orally over 3 consecutive days, while PQ was given over 7 days with a total dosage of 3.5 mg/kg (41).

We obtained the number of *P. vivax* malaria episodes for each participant from the Epidemiological Surveillance System for Malaria (SIVEP-Malaria). Recurrence of *P. vivax* was defined as a new episode that was microscopically diagnosed according to the case report, and it occurred within an interval ranging from 29 to 180 days after the initial episode. In Brazil, the first recurrence commonly occurs within this interval (42). We considered overall recurrence rates due to the inability to distinguish reliably relapse from reinfection or recrudescence by clinical assessment or parasite genotyping (43, 44).

### Blood sample collection and DNA and RNA extraction

In this exploratory study, the number of individuals enrolled was limited by logistic reasons. Blood samples were collected from participants at two time points: on admission (0 hours) before treatment and on the third day (72 hours) after starting antimalarials. Blood samples collected within 72 hours were obtained through active search, where the study team visited the participant’s home to collect the blood sample. This timeframe was selected based on published data indicating that the median time for *P. vivax* gametocyte clearance is approximately 3 days (45, 46). From each patient, 5 mL peripheral blood was drawn during the survey and stored at 4°C in a tube containing the anticoagulant EDTA. The QIAamp DNA Mini Kit (Qiagen) was used following the manufacturer’s instructions for extracting DNA from 200 µL of whole blood, resulting in 50 µL of DNA.

Two 200-µL aliquots of whole blood were collected with 1 mL of RNAprotect (Qiagen) added to each sample for RNA extraction. The samples were then stored at −20°C until transportation to the René Rachou Institute laboratory in Belo Horizonte, where they were kept at −80°C until extraction. RNA extraction was performed using the RNeasy Plus Mini Kit (Qiagen) according to the manufacturer’s instructions, with both blood aliquots processed together. Pellets from both samples were combined to proceed with the extraction. To remove any trace of DNA contamination during RNA extraction, DNA digestion was performed using the commercial Turbo DNA-free Kit (Invitrogen) according to the manufacturer’s protocol.

### CYP2D6 polymorphism genotyping and copy number analysis

Eight single-nucleotide polymorphisms (SNPs) (C1584G, C100T, C1023T, G1846A, C2580T, G2988A, G3183A, and G4180C) and one deletion (2615_2617delAAG) in the *CYP2D6* gene and the copy number of the gene were assayed by qPCR. We used specific hydrolysis probes for each polymorphism (TaqMan SNP genotyping assays; Thermo Fisher Scientific). All amplification reactions were performed according to the protocol described by Silvino and colleagues (15). Amplification and fluorescence detection were carried out using the ViiA 7 real-time PCR system (Thermo Fisher Scientific). The results were analyzed by QuantStudio real-time PCR software (Thermo Fisher Scientific) and the Thermo Fisher cloud platform (Thermo Fisher Scientific).

The copy number of the *CYP2D6* gene was determined by quantitative PCR (qPCR) using the Hs00010001_cn assay (Thermo Fisher Scientific) for gene deletion and amplification detection. All amplification reactions were performed according to the protocol described by Silvino and colleagues (15). Amplification and fluorescence detection were carried out in the ViiA 7 real-time PCR system (Thermo Fisher Scientific), and the number of copies was estimated in CopyCaller v.2.0 software. Only samples with confidence values greater than 95% and absolute z-scores of <1.75 were considered in our analysis.

### Prediction of the *CYP2D6* phenotype based on the AS model

The inferred haplotypes were compared to the *CYP2D6* haplotypes derived from the Pharmacogene Variation Consortium (PharmVar) database for allele designation. Haplotypes that did not match known *CYP2D6* alleles were designated undetermined. For each allelic variant, the value of CYP2D6 metabolic activity relative to that of the fully functional CYP2D6*1 reference allele was assigned. A value of 1 was assigned to the fully functional reference CYP2D6*1 allele, and a value of 0 (zero) was assigned to nonfunctional alleles. Reduced activity alleles received a value of 0.5 to reflect the perceived level of activity reduction. Alleles carrying gene duplications or multiplications received double the value compared to that assigned to an allele with a single gene copy. The sums of these values permitted us to classify the subjects as poor metabolizers [gPM (the prefix “g” indicates that the CYP2D6 phenotype was predicted from genotype data); AS of 0], intermediate metabolizers (gIM; AS of 0.25–0.5), normal–slow metabolizers (gNM-S; AS of 1), normal–fast metabolizers (gNM-F; AS of 1.5 and AS of 2), and ultrarapid metabolizers (gUM; AS of >2).

### CPR polymorphism genotyping

The genotyping of A503V (rs1057868), which changes GCC to GTC at codon 1508 in the gene that encodes *CPR*, was assayed by qPCR. We used a specific hydrolysis probe for the target region (Assay ID C___8890131_30; Thermo Fisher Scientific). All amplification reactions were performed in 384-well plates in a total volume of 5 µL in the presence of 2.5 µL 2 × TaqMan universal PCR master mix (Thermo Fisher Scientific), 0.25 µL TaqMan SNP genotyping assay reagent (Thermo Fisher Scientific), 1.25 µL ultrapure water (free of nucleases), and 1 µL target DNA (**≅** 10 ng/ µL). A negative control was prepared containing no template DNA, and heterozygous and homozygous samples were used as positive controls. The thermocycling conditions were initial denaturation at 95°C for 10 min, followed by 50 cycles of 15 sec at 92°C and 90 sec at 60°C, and one cycle of 30 sec at 60°C. Amplification and fluorescence detection were carried out using the ViiA 7 real-time PCR system (Thermo Fisher Scientific). The results were analyzed by QuantStudio real-time PCR software (Thermo Fisher Scientific) and the Thermo Fisher cloud platform (Thermo Fisher Scientific).

### Quantification of *P. vivax* gametocytes by qPCR

We obtained complementary DNA (cDNA) through reverse transcription (RT) that was performed using the SuperScript IV Reverse Transcriptase (Invitrogen, Life Technologies) together with random primers according to the manufacturer’s protocol, except for the volume used of SuperScript IV (0.5 µL was used in this study). All reactions were performed in a Veriti 96-well Thermal Cycler (Thermo Fisher Scientific).

The quantification of gametocytes was performed using plasmids containing fragments of interest. For that, we used pGEM-T Easy Vector (Promega) and linearized the plasmids with ApaI enzyme (Promega). The primer sequences used to amplify the *18 s rRNA* and *pvs25* targets were previously described by Wampfler and colleagues (47). *pvs25* qPCR experiments were performed using the following protocol: 5 µL of GoTaq qPCR Master Mix (Promega), 200 nM of primers, 3.6 µL of ultrapure water (free of nucleases), and 1 µl of cDNA (**≅** 200 ng). The quantification of the *18 s rRNA* gene was performed using 5 µL of GoTaq qPCR Master Mix (Promega), 900 nM of each primer, 2.2 µL of ultrapure water (free of nucleases), and 1 µL of diluted cDNA (**≅** 2 ng). As this gene is present in greater abundance, the cDNA samples were diluted 100 x for the quantification of this target. The cycling parameters used for the quantification of gametocytes are the following: 2 min at 95°C and 40 cycles of 15 sec at 95°C and 1 min at 60°C, followed by a melt curve analysis. All amplification and fluorescence detection procedures were performed on the ViiA7 Real-Time PCR System (Applied Biosystems). Quantification results were analyzed in QuantStudio Real Time PCR Software v1.3.7. All samples were analyzed in triplicate in 384-well plates. Replicates with standard deviation >0.3 were omitted from the analysis.

The limit of detection (LOD) and the limit of quantification (LOQ) for the *pvs25* assay were estimated from five-fold serial dilutions of cDNA ranging from 4 ng/μL to 0.007 ng/μL with five replicates for each dilution point, following the methodology proposed by Forootan and colleagues (48). The *pvs25* assay was performed with an efficiency of 87% (R^2^ = 0.993). The LOD for *pvs25* was equal to 0.045 copies/uL and the LOQ was equal to 0.174 copies/μL.

### Measurement of primaquine levels

The measurement of primaquine levels was conducted on blood samples collected at 72 hours during the treatment. Primaquine was measured in a reverse-phase HPLC system with UV detection (Flexar, Perkin Elmer, Shelton, MA US), after a previous step of separation of the filter paper following by liquid–liquid extraction with methyl-ter-butyl-ether in alkaline media (49). The column was an RP-18 (X terra 4.6  ×  150 mm, i.d. 5 µm) at 25°C, and the mobile phase was methanol and formic acid (1:3) eluted at a flow rate of 1.0 mL/min and monitored at 263 nm. The limit of detection was 15 ng/mL, and the limit of quantification was 25 ng/mL. The assay was linear from 25 to 2,000 ng/mL and the intra- and inter-assay mean coefficients of variation were 12.5% and 15.5%, respectively. The mean recovery was 82% for the parent compound. Chloroquine, desethylchloroquine, mefloquine, carboxy-mefloquine, or acetaminophen did not interfere in the detection of the analyte.

### Statistical analysis

Proportions are given with 95% confidence intervals and compared with χ^2^ test or Fisher’s exact test. Comparison of continuous variables was performed with the Mann–Whitney test or Kruskal–Wallis test, with Dunn’s post hoc test, as appropriate. The association between two variables was estimated using Spearman’s rank correlation test. The time to the first *P. vivax* malaria recurrence for the CYP2D6 activity level groups was estimated with Kaplan–Meier survival analysis and compared using the log-rank test. All tests were two-sided, and a *P*-value less than 0.05 was considered statistically significant. We used the generalized estimating equations (GEEs) approach to examine the effects of CYP2D6/CPR mutation on gametocyte densities within 72 hours of treatment. We included the relevant covariates, such as age (as a measure of exposure to malaria), *18* s rRNA transcript levels, CYP2D6 predicted activity, and CPR status. Linear regression modeling (LRM) was performed to explain the relationship between PQ blood levels and the covariates: gametocyte densities, *18 s rRNA* transcript levels, CYP2D6 predicted activity, and CPR status. To ensure the validity of our analysis, we assessed the assumptions of the LRM. We examined residual plots and Q-Q plots to evaluate the linearity, independence, homoscedasticity, and normality assumptions for model residuals. Appropriate adjustments or transformations were applied to meet these assumptions in case of any violations. Covariates were selected for inclusion in the GEE and LRM models if they were associated with the outcome at the 15% level of significance in exploratory unadjusted analysis. The models GEE and LRM were fitted using, respectively, the *geeglm* (geepack) and *lm* functions in R. Statistical analysis was performed using STATA v.14 software, GraphPad Prism version 8.0.2 (GraphPad Software, San Diego, California/USA), and the R v.4.1.1 package.

## SUPPLEMENTAL MATERIAL

Supplemental material aac.01204-23-s0001.pdf Supplemental tables and figures.

ASM does not own the copyrights to Supplemental Material that may be linked to, or accessed through, an article. The authors have granted ASM a non-exclusive, world-wide license to publish the Supplemental Material files. Please contact the corresponding author directly for reuse.
