# Supplementary material for: Delayed gametocyte clearance in Plasmodium vivax malaria is associated with polymorphisms in the cytochrome P450 reductase (CPR)
Source: Antimicrob Agents Chemother. 2024 Feb 27;68(4):e01204-23. doi: 10.1128/aac.01204-23 (PMC10989009; doi:10.1128/aac.01204-23)
Supplement: Supplemental material — Supplemental tables and figures. [file aac.01204-23-s0001.pdf]

SUPPLEMENTARY MATERIAL

SUPPLEMENTARY TABLES

Supplementary Table 1. Frequencies of *CYP2D6* Diplotypes in the study population.

CYP2D6 allele	CYP2D6 activity [a]	Total (n)	Total per activity [b] (n)
*1	Normal	0.355 (71)	0.73 (146)
*2/*35 [c]	Normal	0.335 (67)	
*34	Normal	0.035 (7)	
*39	Normal	0.005 (1)	
*2x*N*	Incr	0.01 (2)	0.01 (2)
*9	Decr	0.02 (4)	0.10 (20)
*10	Decr	0.005 (1)	
*41	Decr	0.03 (6)	
*29	Decr	0.045 (9)	
*4	None	0.095 (19)	0.15 (30)
*5	None	0.055 (11)	
Others [d]	ND	0.01 (2)	0.01 (2)

n = number of chromosomes, n = 200.

[a] CYP2D6 metabolic activity according to Pharmvar. Decr, decreased; Incr, increased; ND, not determined.

[b] Sum of allele frequencies by CYP2D6 activity.

[c] We could not differentiate alleles *2 (normal) and *35 (normal) using SNP panel genotyped here.

[d] The SNP combination did not match the known CYP2D6 allele, or the duplications could not be unambiguously assigned to an allele in some heterozygous individuals.

Supplementary Table 2. Association between CPR status and gametocyte clearance in *P. vivax* malaria.

Variable	β coefficient (95% CI) [a]	*P*	Adjusted *P* value [b]
Total parasitemia (18S rRNA)	0.590 (0.416 – 0.764)	**< 0.001**	**< 0.001**
Age	-0.024 (-0.059 – 0.011)	0.182	0.218
CPR Mutated	-1.614 (-3.295 – 0.068)	0.060	0.090
CYP2D6 Impaired	-0.426 (-1.382 – 0.531)	0.383	0.383
Time	-11.674 (-14.087 – -9.262)	**< 0.001**	**< 0.001**
CPR Mutated x Time	4.054 (0.848 – 7.260)	**0.013**	**0.026**

[a] Generalized Estimating Equation (GEE) was adjusted to evaluate the association between CPR status and gametocyte clearance over 3-day follow-up, controlled by the effect of potentially confounding variables, such as the total parasitemia (asexual and sexual stages), age, and CYP2D6 phenotype. The final model was based on 89 subjects. Bold values represent statistically significant results.

[b] Benjamini-Hochberg Adjusted *P* value at $q = 0.05$.

Supplementary Table 3. Association between PQ blood level and gametocyte clearance in *P. vivax* malaria.

Variable	β coefficient (95% CI) [a]	*P*	Adjusted *P* value [b]
Gametocyte density [c]	-0.002 (-0.0027; -0.0004)	**0.010**	**0.038**
CYP2D6 Impaired	-20.8 (-62.0; 20.3)	0.325	0.325
CPR Mutated	-47.5 (-91.0; -4.1)	**0.036**	**0.047**
CYP2D6 Impaired x CPR Mutated	87.5 (16.6; 158.3)	**0.019**	**0.038**

[a] Multiple linear regression analysis of variables associated with PQ blood levels in *P. vivax* malaria. The difference in levels of *18s rRNA* transcripts over time was included in the model, but it was not statistically significant. The final model was based on 66 subjects. Bold values represent statistically significant results.

[b] Benjamini-Hochberg Adjusted *P* value at $q = 0.05$.

[c] Difference in levels of *pvs25* transcript between D3 and D0.

SUPPLEMENTARY FIGURES

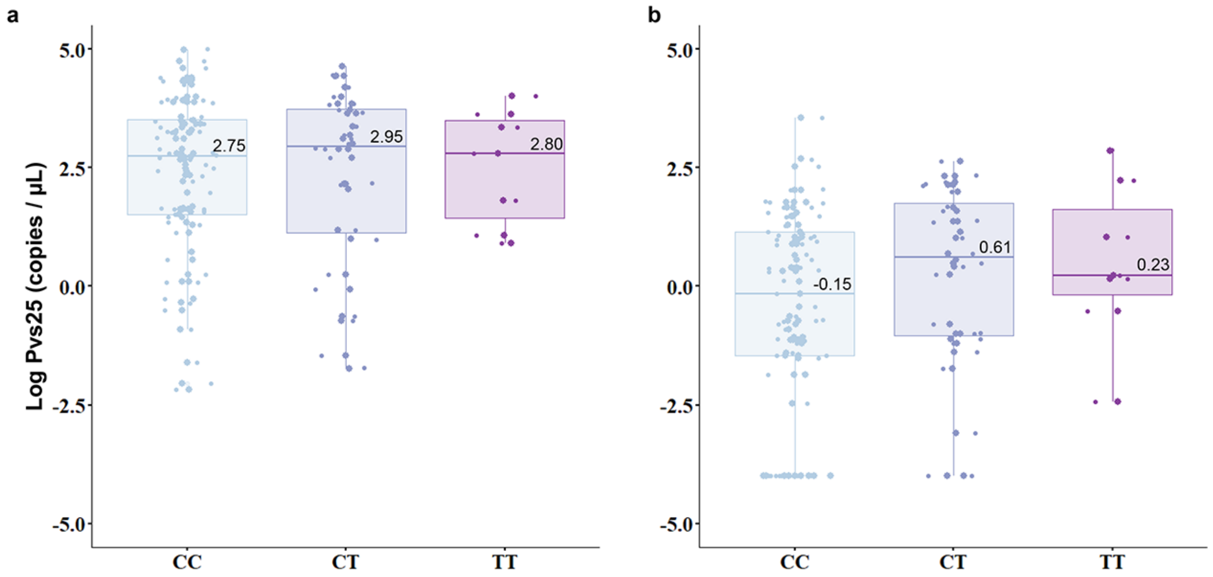

Supplementary Figure 1. Gametocyte density estimates according to genotypes of A503V polymorphism in Cytochrome P450 reductase (CPR). Gametocyte density is shown as the log of *pvs25* transcript levels at (**a**) baseline [median CC = 2.75 (IQR, 1.52-3.51), CT = 2.95 (1.13-3.74), TT = 2.80 (1.43-3.49), *P* = 0.97 by Kruskal-Wallis] and (**b**) 72h after the initial of the treatment [median CC = -0.15 (IQR, -1.46-1.14), CT = 0.61 (-1.03-1.75), TT = 0.23 (-0.18-1.63), *P* = 0.22 by Kruskal-Wallis].

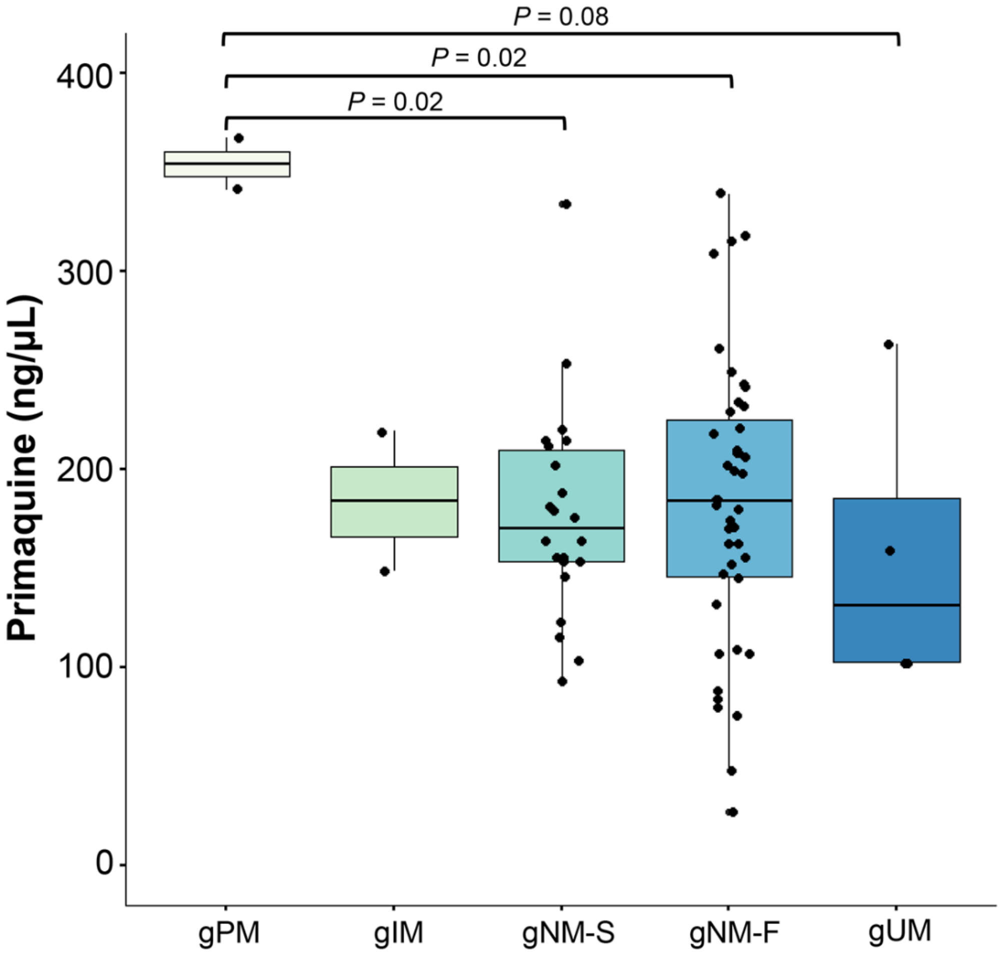

Supplementary Figure 2. Primaquine concentration in whole blood according to CYP2D6 status. Primaquine was measured on day three after the initial treatment. gPM: poor metabolizer; gIM: intermediate metabolizer; gNM-S: normal-slow metabolizer; gNM-F: normal-fast metabolizer; gUM: ultrarapid metabolizer. The median of PQ concentration (and IQR) in ng/µL = 354 (347-361) for gPM, 183 (166-201) for gIM, 170 (153-210) for gNM-S, 184 (146-225) for gNM-F and 131 (103-185) for gUM. The comparison of means was performed by Welch's t-test. *P*-values were adjusted with Holm–Bonferroni's method.
